# Agreement and Reliability of a Newly Developed Tidal Volume Monitoring Device in Bag-Valve Ventilation and Intubation Scenarios: A Simulation-Based Study

**DOI:** 10.3390/bioengineering13080930

**Published:** 2026-08-17

**Authors:** Yoonsuk Lee, Eun Young Lee, Hee Young Lee

**Affiliations:** 1 Department of Emergency Medicine, Wonju College of Medicine, Yonsei University, Wonju 26426, Gangwon State, Republic of Korea; yslee524@gmail.com; 2 Research Institute of Hyperbaric Medicine and Science, Wonju College of Medicine, Yonsei University, Wonju 26426, Gangwon State, Republic of Korea; 3 Office of Medical Research Affairs, Yonsei Wonju Health System, Wonju 26427, Gangwon State, Republic of Korea; eyleeys@yonsei.ac.kr

**Keywords:** manual ventilation, tidal volume monitoring, operator-related variability, simulation-based study

## Abstract

**Background**: Manual bag-valve ventilation is a fundamental intervention in prehospital and emergency care but remains highly operator-related and frequently associated with inappropriate tidal volume delivery. Excessive or insufficient ventilation may adversely affect hemodynamics and clinical outcomes. Although real-time tidal volume monitoring has the potential to improve ventilation quality, its measurement performance across different airway conditions has not been sufficiently evaluated. **Methods**: This simulation-based repeated-measures study evaluated the measurement agreement and reliability of a newly developed tidal volume monitoring device during manual ventilation using an adult airway management manikin. Twenty emergency medical technicians performed bag-valve ventilation across three airway scenarios: face mask ventilation, endotracheal tube (ETT) intubation, and supraglottic airway (I-gel) insertion. For each scenario, 10 repeated breaths were recorded. Tidal volumes measured by the test device were compared with simulator-derived reference values. Measurement agreement was assessed using paired *t*-tests, Pearson correlation coefficients, effect sizes, and Bland–Altman analysis, while measurement reliability was evaluated using intraclass correlation coefficients (ICC). Mixed repeated-measures ANOVA was performed to examine interaction effects between airway scenario and operator characteristics. **Results**: The test device consistently overestimated tidal volume compared with the simulator reference across all airway scenarios (*p* < 0.001). Correlation between measurements was weak during mask ventilation (r = 0.174) but strong during ETT (r = 0.854) and I-gel (r = 0.709) ventilation, whereas Bland–Altman analysis demonstrated wider limits of agreement in mask ventilation and narrower limits under intubated conditions. ICC analysis revealed airway-dependent reliability, with minimal agreement in normal BVM ventilation but substantially higher agreement during ET-tube and I-gel intubation. Significant effects of airway scenario and operator characteristics (gender, age group, and clinical experience level) were observed, with notable interaction effects between airway scenario and clinical experience. **Conclusions**: Despite systematic overestimation, the tidal volume monitoring device demonstrated consistent and repeatable performance, particularly under secured airway conditions. Its primary clinical value may lie in reducing operator-related variability and supporting safer manual ventilation through real-time feedback, rather than replacing gold-standard measurement systems. Further algorithm refinement and clinical validation are warranted.

## 1. Introduction

Effective ventilation is a cornerstone of cardiopulmonary resuscitation (CPR) and emergency care, particularly during the critical window of patient transport outside the hospital. Manual bag-valve-mask (BVM) ventilation remains a widely used method in such prehospital contexts due to its portability, rapid deployment, and lack of requirement for complex infrastructure. However, the effectiveness and safety of manual BVM ventilation are profoundly influenced by the skill and technique of the operator. Inaccurate delivery of tidal volume, either through excessive ventilation (hyperventilation) or insufficient ventilation (hypoventilation), can lead to serious consequences, such as secondary lung injury, compromised oxygenation, and worsened clinical outcomes.

Manual resuscitators lack mechanisms to regulate tidal volume, resulting in substantial variability in delivered ventilation even among experienced healthcare providers. For instance, Wenzel et al. reported that it is possible for even experienced anesthesiologists to perform ventilation with excessively short inspiratory times or overly large tidal volumes, which may result in stomach inflation and associated risks such as regurgitation, aspiration, and serious pulmonary injury [1]. Similarly, evaluations of ventilation performance in manikin studies have frequently highlighted non-compliance with guideline-recommended volumes and rates across a range of providers, with one systematic review noting that ventilation in simulated out-of-hospital cardiac arrest rarely meets guidelines [2]. In South Korea, out-of-hospital cardiac arrest (OHCA) remains a significant public health concern, with a one-year survival rate of approximately 8.2% [3]. This survival rate is notably lower compared to gains observed in other regions, despite global efforts to improve prehospital care [4]. A comprehensive registry analysis indicates that although survival outcomes have improved over time, disparities based on regional characteristics persist, underscoring an urgent need for enhanced resuscitation strategies [5].

Despite being a life-saving technique, BVM ventilation is subject to significant physiological risks when improperly administered. Specifically, manual hyperventilation increases intrathoracic pressure, reduces venous return, and may precipitate gastric insufflation, compromising hemodynamics and increasing patient morbidity [6,7]. Gastric inflation, in turn, exacerbates these risks by further impairing ventilation and circulation, potentially leading to aspiration or diminished cardiac output [8]. Given that manual resuscitators inherently lack tidal volume control, such complications are pervasive across user clinical experience levels, making over-inflation a commonly observed issue in manual ventilation. To address these critical shortcomings, a reliable tidal volume monitoring system capable of delivering real-time feedback is essential. Although commercially available tidal volume monitoring systems are capable of accurately measuring respiratory parameters, most are designed for integration with mechanical ventilators, anesthesia workstations, or stationary respiratory monitoring systems. Consequently, they are not readily applicable during manual bag-valve ventilation in emergency, prehospital, or out-of-hospital settings, where portability, rapid deployment, and seamless integration into existing resuscitation workflows are essential. Such a device has the potential to reduce operator dependency and variability, improve ventilation agreement, and minimize lung injury from improper pressure or volume. Although commercially available tidal volume monitoring systems provide accurate respiratory measurements, they are primarily intended for use with mechanical ventilators, anesthesia workstations, or stationary respiratory monitoring systems. Consequently, they generally require dedicated respiratory circuits or integrated monitoring platforms, making their application during manual bag-valve ventilation in emergency or prehospital settings impractical. Moreover, if designed for accessibility and user-friendliness, they could be deployed across diverse settings, including ambulances, community health centers, and public spaces, thereby elevating emergency care quality and patient survival. In alignment with this need, the present simulation-based study was designed to evaluate a newly developed tidal volume monitoring device for BVM ventilation. Unlike conventional respiratory monitoring systems that are primarily designed for mechanical ventilators or intensive care settings, the proposed device was developed specifically for manual bag-valve ventilation. The engineering novelty of the device lies in the integration of a compact digital flow sensor, real-time signal processing, and intuitive visual feedback into a portable platform that can be seamlessly incorporated into existing manual resuscitation workflows without requiring modification of conventional bag-valve equipment. By providing immediate quantitative feedback on delivered tidal volume, the system addresses the long-standing technological limitation of subjective ventilation assessment during manual resuscitation.

This study aims to compare the device’s measurements with simulator-derived reference values under controlled conditions to assess measurement agreement, evaluate its reliability and repeatability across varying operator and airway conditions, and examine its potential to reduce operator-related variability during manual ventilation. Establishing measurement agreement under representative airway conditions was considered a prerequisite for future studies evaluating the clinical effectiveness of real-time tidal volume feedback during manual ventilation.

## 2. Materials and Methods

### 2.1. Study Design

This study was designed as a simulation-based method-comparison study using a repeated-measures design to evaluate the effectiveness of a tidal volume monitoring device during manual bagging in three scenarios, including bag-valve-mask ventilation, endotracheal tube (ETT) intubation, and supraglottic airway device (I-gel) intubation, on an adult airway management training manikin. The required sample size was calculated using G*Power version 3.1.9.7 (Heinrich Heine University, Düsseldorf, Germany), assuming an effect size of 0.85 for Pearson correlation analysis to evaluate the association between tidal volume measurements obtained from the proposed device and the simulator-based comparative measurement system [9,10]. Correlation analysis was selected because the present study was designed not only to assess measurement agreement but also to evaluate the relationship, reliability, and operator-related performance of the proposed device across multiple airway scenarios. Based on these assumptions, a minimum sample size of 18 participants was required. To account for an anticipated dropout rate of approximately 10%, a total sample size of 20 participants was enrolled. The study involved a single group of 20 emergency medical technicians working at the Regional Emergency Medical Center at Wonju Severance Christian Hospital, who participated in the comparative evaluation of the newly developed device. All participants provided written informed consent before participating in the study. This clinical study complied with the International Conference on Harmonization (ICH) Guidelines and the principles of the Declaration of Helsinki. The study protocol received institutional review board approval prior to participant recruitment (IRB approval number: CR223014). The study was subsequently registered with the Clinical Research Information Service (CRIS; Trial Registration No.: KCT0011169), a primary registry of the World Health Organization International Clinical Trials Registry Platform (ICTRP).

### 2.2. Equipment

#### 2.2.1. Test Device: Newly Developed Tidal Volume Monitoring Device

**Figure 1:**
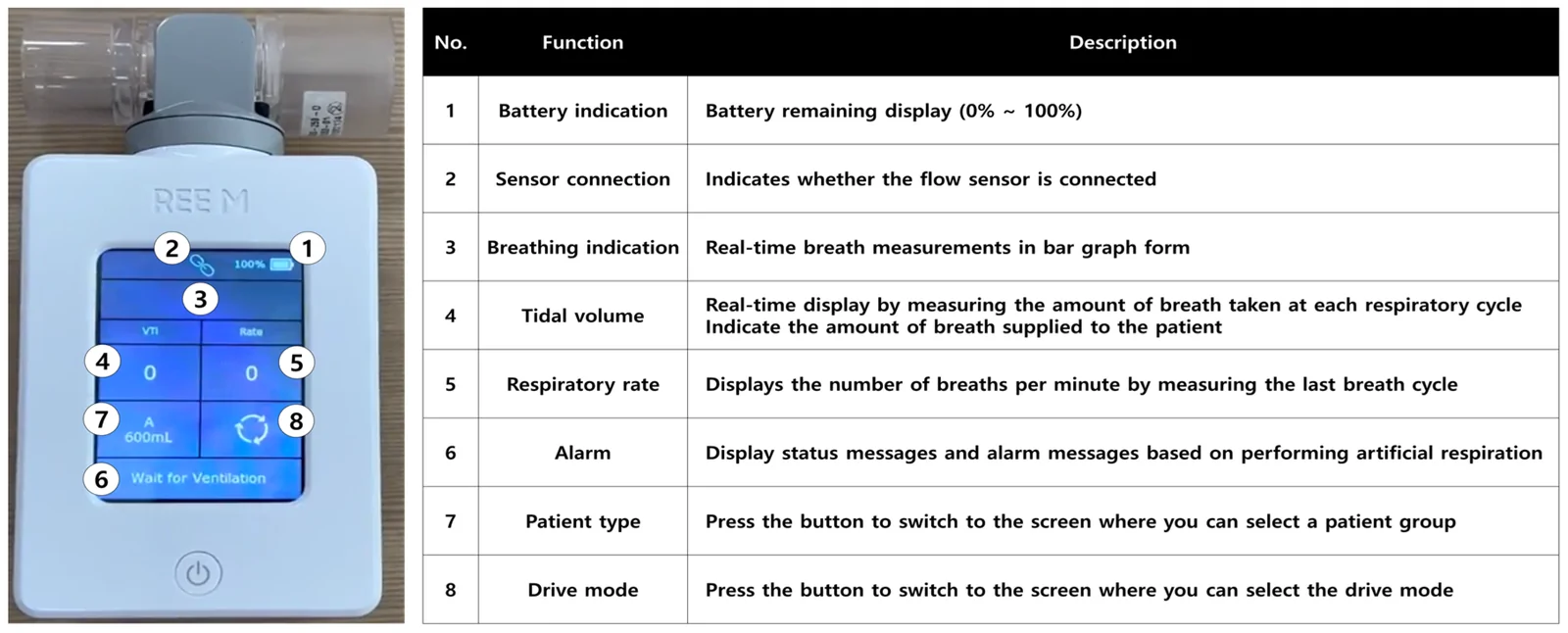
Photos of the tidal volume monitoring device and an explanation of the monitoring screen.

The newly developed tidal volume monitoring device (test device) was specifically designed to address the lack of objective tidal volume monitoring during manual bag-valve ventilation. Unlike conventional respiratory monitoring systems that rely on mechanical ventilators or stationary respiratory monitors, the proposed device incorporates a compact digital flow sensor capable of measuring airflow and calculating tidal volume in real time. The calculated tidal volume is immediately displayed to the user through an intuitive visual interface, providing real-time feedback that supports adherence to recommended ventilation practices during manual ventilation. This integrated system architecture enables continuous quantitative monitoring without interrupting conventional manual ventilation procedures or requiring additional complex equipment. The device is designed to measure and visualize the volume of air delivered to a patient via a manual resuscitator. The disposable digital flow sensor is positioned between the manual resuscitator and the patient interface using standard ISO 22 mm/15 mm respiratory connectors. According to the manufacturer’s specifications, the system measures tidal volumes over a range of 50–1800 mL with a display resolution of 1 mL and a specified measurement accuracy of ±10%. As air flows through the digital flow sensor, real-time airflow data are transmitted to an embedded respiratory processing module, where tidal volume is continuously calculated and displayed on the monitor. As shown in Figure 1, the monitoring screen includes several key indicators: battery status, sensor connection status, real-time breathing visualization, tidal volume per breath, respiratory rate per minute, ventilation status messages, patient type selection (Adult, Pediatric, Infant), and drive mode options (CPR 30:2/15:2, Continuous). The bar graph dynamically reflects each breath cycle, increasing during inhalation and decreasing during exhalation based on the measured air or oxygen volume. The color-coded bar graph presented yellow for insufficient volume, green for adequate volume, and red for excessive volume, allowing users to assess ventilation quality at a glance (③ Breathing indication in Figure 1). The device was factory calibrated before delivery according to the manufacturer’s calibration procedure. No additional calibration was performed before individual experimental sessions because all experiments were conducted using the same device under identical laboratory conditions, thereby ensuring consistent measurement conditions throughout the study.

#### 2.2.2. Comparative Device: An Adult Airway Management Training Simulator

In this study, an adult airway management training simulator (BT-CSIE^®^, BT Inc., Goyang, Gyeonggi, Republic of Korea) was employed to provide a standardized and reproducible environment for practicing airway interventions. This high-fidelity upper torso simulator replicates realistic anatomical features and tactile feedback, enabling participants to perform essential airway maneuvers, including head-tilt/chin-lift, sniffing position, and jaw thrust techniques, as well as manual ventilation using a bag-valve-mask (BVM). The simulator supports the insertion of oral and nasal airway devices, endotracheal tubes (ETT), and supraglottic airway devices such as laryngeal mask airways (LMA) and Combitubes, while also facilitating laryngoscope-guided intubation practice. Functional feedback mechanisms are incorporated to provide visual confirmation of chest expansion during pulmonary inflation, abdominal distension during gastric insufflation, and misplacement detection such as esophageal or right main bronchial intubation. Additional features include warning signals for excessive laryngoscope pressure indicating potential dental injury, and performance indicators for proper airway maneuvers. Moreover, the simulator integrates an internal volume measurement sensor and a tablet-based color touchscreen that delivers real-time feedback, training data visualization, and logging functions. In the present study, the simulator was used as a standardized comparative measurement system to evaluate measurement agreement under controlled simulation conditions rather than as a certified laboratory reference standard. Although the simulator provides consistent and reproducible tidal volume measurements for training and research purposes, its integrated monitoring system does not represent a pneumotachograph- or spirometry-based gold standard for absolute respiratory volume measurement. Wireless Bluetooth connectivity, adjustable scoring algorithms, and dual AC/battery power supply ensure flexible use in various training environments.

#### 2.2.3. Airway Adjunct Devices: BVM, ETT, I-Gel

**Figure 2:**
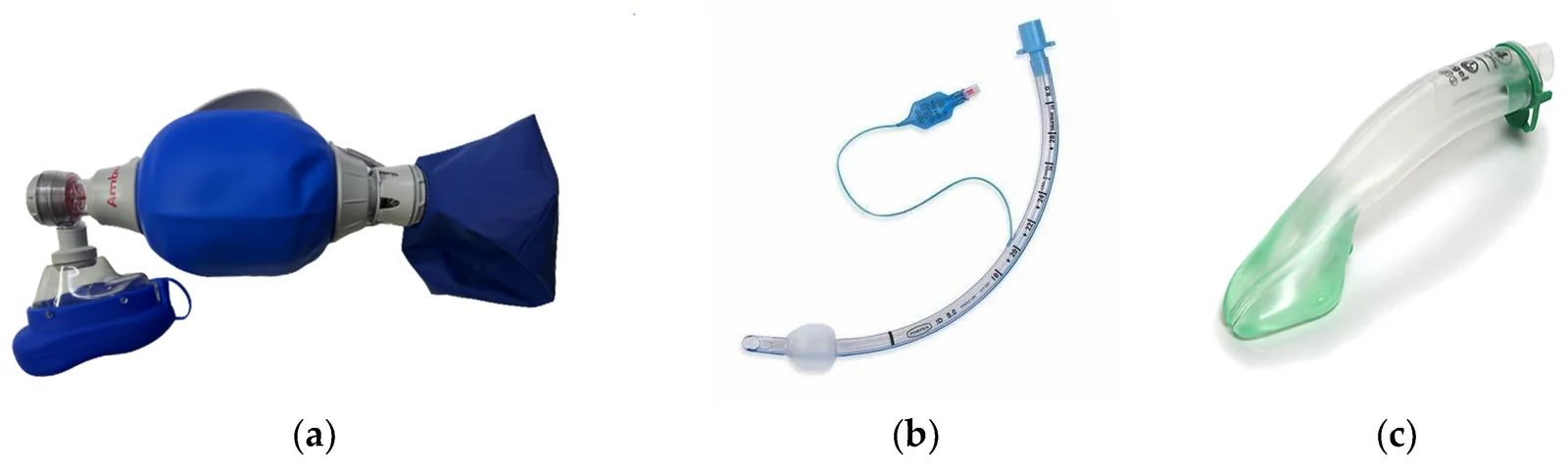
Three airway adjunct devices: (**a**) BVM, (**b**) ETT, and (**c**) I-gel.

As shown in Figure 2, three airway adjunct devices were utilized to simulate different ventilation scenarios in this study: a bag-valve-mask (BVM; Ambu^®^ Mark IV, Ambu A/S, Ballerup, Denmark), an endotracheal tube (ETT; Cuff 6.5, Hyupsung Medical Co. Ltd., Yangju, Gyeonggi, Republic of Korea), and a supraglottic airway device (I-gel; 8204000, Insung Medical Co. Ltd., Wonju, Gangwon State, Republic of Korea). The BVM is a manual ventilation device commonly employed in prehospital and emergency settings, allowing operators to deliver positive-pressure ventilation to patients without an advanced airway. The ETT provides a secured airway through endotracheal intubation, ensuring controlled ventilation and protection against aspiration, and is considered the clinical standard for definitive airway management. The I-gel, a second-generation supraglottic airway device, offers rapid insertion and a non-inflatable cuff design to establish an effective airway with minimal training, serving as an intermediate option between BVM and ETT. Collectively, these devices represent a spectrum of airway management techniques, from manual ventilation to advanced airway interventions, enabling evaluation of the tidal volume monitoring device across varying operational conditions and airway complexities.

### 2.3. Clinical Trial Protocol

This study compared device performance during mask ventilation (BVM) and advanced airway management (ETT and I-gel) using the same participants. Before formal data collection, all participants completed a standardized orientation session and received structured training on the operation of the newly developed tidal volume monitoring device and the applied airway models using audiovisual educational materials to ensure familiarity with the device and study procedures. A washout period of approximately 10 min was provided between the training session and the formal evaluation to minimize the immediate influence of training while simulating a realistic clinical environment. The order of the three airway management scenarios (BVM, ETT, and I-gel) was randomized for each participant to minimize potential order effects and learning bias associated with repeated measurements. Participants were assessed using predefined scenarios (10 breaths per minute) tailored to each airway model (BVM, ETT, I-gel) [11]. Across all scenarios, the flow sensor was installed at the same relative position: it was attached to the main body of the test device, with the outlet side connected to the Ambu bag and the inlet side linked to each airway adjunct (BVM, ETT, or I-gel). This standardized configuration ensured identical sensor placement and measurement conditions for all airway types, thereby minimizing positional bias in tidal volume assessment. Observers monitored participants’ performance and recorded measured ventilation values. All sessions were recorded for post-hoc error analysis.

### 2.4. Statistical Analysis

Statistical analyses were conducted using SPSS Statistics (Version 28.0, IBM Corp., Armonk, NY, USA). All statistical analyses were conducted to evaluate the linear association, measurement agreement, and reliability of tidal volume measurements across three airway management scenarios—Mask ventilation, endotracheal intubation (ET-tube), and supraglottic airway insertion (I-gel)—using a bag-valve-mask (BVM) device. Measurements were obtained from two sources: the embedded software of the adult airway management training simulator (BT-CSIE^®^), and the newly developed tidal volume monitoring device attached to each airway interface. A total of 20 participants performed 10 repetitions per scenario, resulting in 600 data points (20 participants × 3 scenarios × 10 repetitions). Descriptive statistics were used to summarize the central tendency and distribution of tidal volume for each airway device and measurement source, including mean ± standard deviation, median with interquartile range, and minimum and maximum values. These metrics were also stratified by participant characteristics such as sex, age group (<25, 25–29, ≥30), and clinical experience level (≤12 months, 13–60 months, >60 months) to explore subgroup differences. Pearson correlation analysis was performed to evaluate the strength of the linear association between tidal volume measurements obtained from the newly developed tidal volume monitoring device and the simulator-based comparative measurement system. Measurement agreement between the two systems was assessed using Bland–Altman analysis by calculating the mean bias and the 95% limits of agreement. For clinical interpretation, adult ventilation guidelines from the American Heart Association (AHA) and the European Resuscitation Council (ERC) recommend delivering approximately 500–600 mL tidal volume during manual bag-valve ventilation. This range corresponds to roughly 6–8 mL/kg for a typical adult and provides a clinically acceptable reference threshold for interpreting Bland–Altman limits of agreement [12,13]. This threshold was used as the reference criterion to determine whether the observed measurement differences between devices were within clinically tolerable ranges. Moreover, agreement between the two measurement systems was quantified using the intraclass correlation coefficient (ICC) calculated from a two-way mixed-effects model with an absolute agreement definition for single measurements [ICC(A,1)]. ICC estimates were reported together with their corresponding 95% confidence intervals. A repeated-measured ANOVA was conducted to compare tidal volume across devices within subjects, with Mauchly’s test applied to assess the assumption of sphericity, and Greenhouse-Geisser correction was used when violated. Where applicable, paired *t*-tests were performed only to determine whether statistically significant mean differences (systematic bias) existed between the two measurement systems within each airway scenario. These tests were not used to evaluate measurement agreement, which was assessed primarily using Bland–Altman analysis. Post-hoc pairwise comparisons were performed using Bonferroni adjustment to identify significant differences between device types. To examine interaction effects between airway device and participant characteristics, a two-way mixed ANOVA was employed, incorporating sex, age group, and clinical experience level as between-subject factors.

## 3. Results

### 3.1. General Characteristics of the Study Population

Table 1 summarizes the baseline characteristics of the study participants. A total of 20 participants were enrolled in the study. Among them, five participants (25.0%) were male and 15 (75.0%) were female. The mean age of the participants was 25.95 years (SD ± 2.89), with a median age of 25 years and an interquartile range (IQR) of 24 to 28 years. Based on age distribution, nine participants (45.0%) were under 25 years, eight participants (40.0%) were between 25 and 29 years, and three participants (15.0%) were aged 30 years or older. The average clinical experience was 36.50 months (SD ± 38.46), with a median of 18.5 months [IQR: 7.5–56]. Participants were categorized into three career experience groups: six participants (30.0%) were classified as beginners (≤12 months), nine (45.0%) as intermediate (13–60 months), and five (25.0%) as experts (>60 months). Regarding prior experience with manual ventilators, 17 participants (85.0%) reported having used one before, while three participants (15.0%) had no prior experience. Most participants (*n* = 17, 85.0%) had prior experience using manual ventilators. Among those with prior experience, the Ambu^®^ Mark IV (Ambu A/S, Ballerup, Denmark) was the most commonly used model (17/17, 100%), while the LSR (Laerdal Medical AS, Stavanger, Norway) and MR-010 (MOW Medical Co., Ltd., Seoul, Republic of Korea) were each used by one participant (1/17, 5.9%).

### 3.2. Comparison of Tidal Volume Between the Training Simulator and the Test Device Across Airway Scenarios

A paired-samples *t*-test was performed to compare tidal volume measurements between the adult airway management training simulator and the test device across three airway scenarios. As shown in Table 2, the test device consistently reported significantly higher tidal volumes than the simulator across all scenarios. In the normal BVM ventilation scenario, the mean difference was 284.61 mL (t(199) = 37.61, *p* < 0.001), with a large effect size (Cohen’s d = 2.66). For the ET-tube intubation scenario, the mean difference was 79.63 mL (t(199) = 25.19, *p* < 0.001; d = 1.78), and for the I-gel tube intubation scenario, the difference was 99.55 mL (t(199) = 25.20, *p* < 0.001; d = 1.78). These results indicate the presence of a statistically significant systematic measurement bias between the two measurement systems but do not, by themselves, indicate measurement agreement. These results indicate a systematic overestimation of tidal volume by the test device compared to the simulator. Pearson correlation analysis revealed only a weak correlation in the BVM ventilation scenario (r = 0.174), but strong correlations in the ET-tube (r = 0.854, *p* < 0.001) and I-gel (r = 0.709, *p* < 0.001) intubation scenarios, suggesting varying levels of agreement depending on the airway technique. These correlation coefficients describe the degree of linear association only and should not be interpreted as measures of agreement.

However, correlation reflects association rather than true agreement; therefore, Bland–Altman analysis was additionally performed to assess measurement agreement. To further quantify measurement agreement beyond correlation and Bland–Altman analysis, intraclass correlation coefficients (ICC) were computed (Table 3). The ICC results revealed poor reliability in the BVM scenario (ICC = 0.025), poor reliability during endotracheal intubation (ICC = 0.486), and poor-to-moderate reliability for the I-gel scenario (ICC = 0.350). These findings align with the observed correlation patterns, emphasizing that measurement reliability between the simulator and the test device varied considerably across airway types—with the highest consistency under sealed endotracheal conditions and the lowest reliability during unsealed mask ventilation.

**Figure 3:**
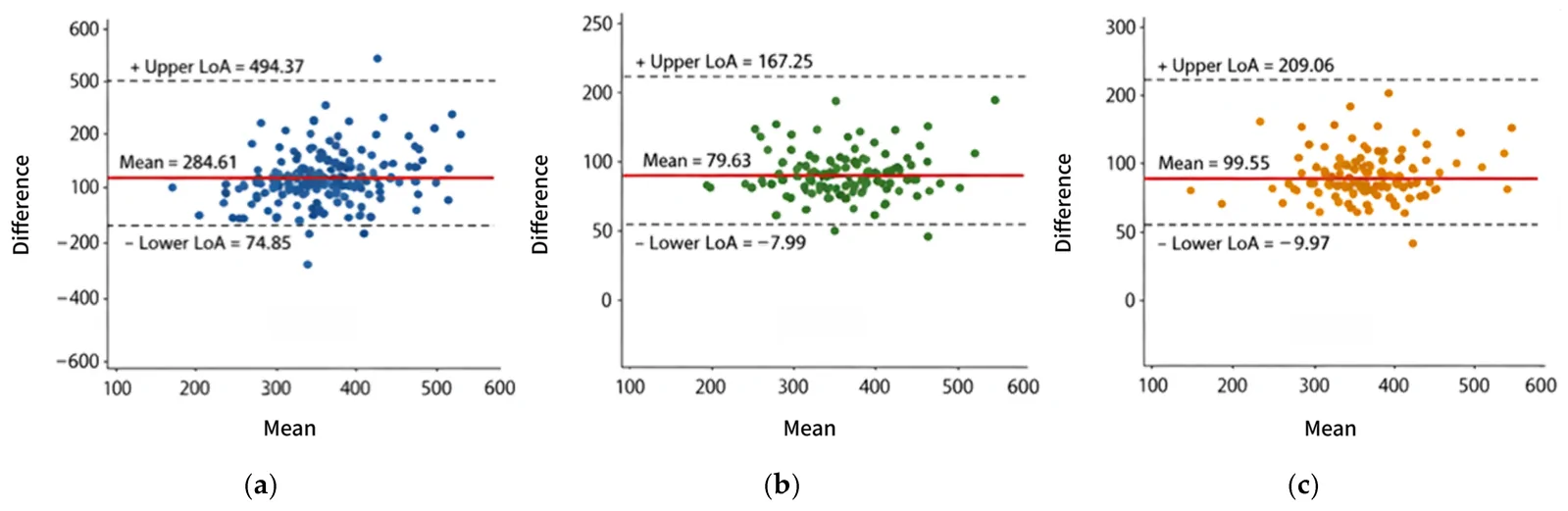
Bland–Altman plots comparing the mean difference (bias) and the 95% limits of agreement between the simulator and the test device across airway scenarios: (**a**) BVM, (**b**) ETT, and (**c**) I-gel. (blue: BVM, green: ETT, orange: I-gel).

In Figure 3, Bland–Altman plots illustrate the agreement between the test device and the training simulator across airway conditions. In the BVM scenario, the mean difference was +284.61 mL, with wide limits of agreement (LoA: 74.85 to 494.37), indicating poor consistency and limited interchangeability under unsealed mask ventilation. In contrast, the ET-tube intubation scenario showed a smaller mean difference (+79.63 mL) and substantially narrower LoA (−7.99 to 167.25), reflecting markedly improved agreement under sealed airway conditions. For the I-gel supraglottic airway scenario, the mean difference was +99.55 mL, with LoA ranging from −9.97 to 209.06, demonstrating moderate agreement that was less robust than ET-tube intubation but clearly superior to mask ventilation.

Overall, the results demonstrate that the test device and the training simulator measurements are most consistent and interchangeable during ET-tube intubation, moderately reliable with I-gel tube intubation, and least reliable in normal airway conditions. These findings highlight the importance of considering airway type when interpreting and comparing tidal volume measurements between devices.

### 3.3. Interaction Effects of Participant Characteristics on Measurement Error Across Airway Scenarios

As shown in Table 4, a mixed repeated measures ANOVA was conducted to examine the interaction effects of participant characteristics (gender, age group, clinical experience level) on the tidal volume discrepancy between BT-CSIE^®^ and the test device across three airway scenarios. Mauchly’s test indicated a violation of the sphericity assumption (W = 0.653, χ^2^ = 80.987, *p* < 0.001), and the Greenhouse–Geisser correction was applied. Significant between-subjects effects were found for gender (F(1,191) = 46.17, *p* < 0.001, ηp2 = 0.195), age group (F(2,191) = 5.03, *p* = 0.007, ηp2 = 0.050), and clinical experience level (F(2,191) = 7.41, *p* < 0.001, ηp2 = 0.072). Regarding within-subjects effects, a strong main effect of scenario was observed (F(1.49,283.58) = 431.40, *p* < 0.001, ηp2 = 0.743), demonstrating that measurement error varied substantially across airway types. Significant interaction effects emerged for scenario × gender (F(1.49,283.58) = 44.31, *p* < 0.001, ηp2 = 0.189) and scenario × clinical experience level (F(2.97,283.58) = 4.60, *p* = 0.004, ηp2 = 0.046), whereas the scenario × age group interaction was not significant (*p* = 0.560). Simple main effects analyses indicated statistically significant differences according to gender and clinical experience level across scenarios. However, given the limited sample size and the exploratory nature of these subgroup analyses, these findings should be interpreted cautiously and considered hypothesis-generating rather than definitive evidence of operator-related performance differences.

In Table 5, post-hoc Bonferroni comparisons revealed that participants with intermediate experience showed significantly lower discrepancy than beginners (mean difference = −28.11, *p* < 0.001), and experts differed significantly from intermediates (mean difference = 19.99, *p* = 0.018), but not from beginners (mean difference = −8.12, *p* = 0.899). Regarding age, only the comparison between the <25 and 25–29 groups reached statistical significance (mean difference = 24.05, *p* = 0.001), while other pairwise comparisons were not significant.

## 4. Discussion

### 4.1. Overall Interpretation of the Study Findings

This simulation-based study evaluated the agreement, reliability, and operator dependency of a newly developed tidal volume monitoring device during manual bag-valve ventilation across three airway scenarios: mask ventilation, endotracheal intubation, and supraglottic airway insertion. The principal findings were threefold. First, the test device systematically overestimated tidal volume compared with the reference simulator in all scenarios. Second, the relationship between measurements obtained from the two systems varied substantially depending on airway type. Pearson correlation analysis demonstrated stronger linear associations under intubated conditions than during mask ventilation. However, linear association alone does not indicate measurement agreement. Accordingly, Bland–Altman analysis and intraclass correlation coefficients were used as the primary measures of agreement and reliability, both of which consistently showed poor agreement in normal mask ventilation but substantially higher agreement during ET-tube intubation and moderate agreement with I-gel intubation. These findings indicate that although the two systems exhibited stronger linear association under sealed airway conditions, true measurement agreement was supported primarily by the narrower limits of agreement and higher ICC values observed in the intubated airway scenarios, whereas the extremely low ICC in mask ventilation confirmed limited interchangeability under unsealed conditions. This distinction is important because strong linear association between two measurement systems does not necessarily imply clinical interchangeability, which should instead be interpreted primarily from Bland–Altman agreement analysis together with measurement reliability. Third, measurement discrepancies were significantly influenced by operator characteristics, particularly gender and clinical experience level, with notable interaction effects according to ventilation scenario. Collectively, these findings suggest that while the device has limitations in absolute agreement, it demonstrates consistent measurement behavior and higher repeatability specifically under controlled airway conditions where ICC values indicate acceptable reliability. Therefore, its primary value may lie in reducing operator-related variability and supporting safer ventilation practices rather than serving as a replacement for reference-grade measurement systems. Importantly, the systematic bias observed in the present study suggests that the device should currently be interpreted as a trend-monitoring tool rather than an absolute measurement instrument. When used to guide relative adjustments in ventilation volume, rather than relying on absolute numeric values alone, such monitoring systems may still provide clinically meaningful feedback that helps prevent extreme hyperventilation or hypoventilation during manual bagging.

From an engineering perspective, the proposed system represents an integrated solution for real-time tidal volume monitoring in manual ventilation, a clinical setting where objective quantitative respiratory monitoring has traditionally been limited. Rather than introducing a fundamentally new sensing principle, the engineering innovation lies in the practical integration of a compact digital flow sensor, real-time tidal volume computation, and intuitive visual feedback into a portable platform specifically designed for manual bag-valve ventilation. By seamlessly integrating these functions into existing resuscitation workflows without requiring additional complex equipment, the system enables continuous quantitative monitoring while preserving conventional ventilation procedures. This engineering approach has the potential to facilitate broader adoption of objective ventilation monitoring in emergency and prehospital settings and to support more standardized manual ventilation practices.

### 4.2. Systematic Overestimation of Tidal Volume: Engineering Interpretation of Systematic Measurement Bias

Across all three ventilation scenarios, the test device consistently reported higher tidal volumes than the simulator reference, with the largest mean discrepancy observed during mask ventilation. This systematic overestimation is not unexpected and has been reported in prior evaluations of flow-sensor-based monitoring systems used in manual ventilation contexts [14,15]. Several mechanisms may explain this phenomenon. First, mask ventilation is inherently prone to air leakage and measurement discrepancies, particularly in settings without airtight sealing or leak compensation, which reduces the agreement of tidal volume measurements by flow sensors and respiratory monitors [15]. This discrepancy likely contributes to the large negative mean difference and weak correlation observed in the BVM scenario (r = 0.174). Studies using respiratory function monitors in neonatal resuscitation and simulated ventilation have demonstrated that increasing mask leak levels significantly alter measured tidal volume, and mask-leak conditions are associated with poorer correlation between delivered and measured tidal volume [16]. Second, manikin-based evaluations of manual ventilation reveal that inherent limitations of simulators and variability in user technique contribute to measurement bias and high variability in tidal volume delivery [2]. From an engineering perspective, several additional factors may also contribute to the observed systematic overestimation. The response characteristics of the digital flow sensor may vary under the rapidly changing airflow conditions generated during manual bag-valve ventilation, particularly during forceful or irregular bag compression. Because tidal volume is estimated by numerically integrating the measured airflow signal over time, small cumulative errors in flow measurement or signal processing may result in a consistent positive bias. Furthermore, nonlinear sensor behavior outside the optimal operating range and assumptions incorporated into the signal-processing algorithm may further influence volume estimation. As a result, flow integration performed without leak compensation or more advanced pressure–flow modeling may lead to overestimation of delivered volume, particularly during rapid or forceful bagging. Importantly, the magnitude of overestimation decreased substantially in intubated airway scenarios, supporting the interpretation that airway sealing and flow containment play critical roles in measurement agreement. In contrast, the larger discrepancies observed during mask ventilation may reflect greater variability in airflow caused by imperfect mask seal integrity, which is inherently more difficult to control than in intubated conditions and may further amplify flow estimation errors. From a technical perspective, systematic overestimation may be mitigated through several engineering strategies, including optimization of sensor recalibration, implementation of leak-compensation algorithms, and integration of pressure–flow modeling to account for airway resistance and compliance during manual ventilation. Because the detailed implementation of the sensing algorithm is proprietary, the precise contribution of each factor could not be determined in the present study. Nevertheless, future engineering studies should investigate these technical components individually to optimize the measurement algorithm and improve the absolute agreement of real-time tidal volume estimation. Beyond improving measurement agreement, the present findings also provide practical engineering insights for future development of manual ventilation monitoring systems. Specifically, the observed systematic bias suggests that future device optimization should focus not only on improving sensor performance but also on refining adaptive signal-processing algorithms, implementing dynamic calibration strategies, and incorporating leak-compensation techniques capable of accommodating variable airflow conditions encountered during manual ventilation. Furthermore, integration of pressure–flow modeling and intelligent calibration algorithms may enhance measurement robustness across diverse airway conditions and clinical environments. Collectively, these engineering improvements may enhance the agreement, robustness, and clinical applicability of future generations of real-time tidal volume monitoring systems, particularly in emergency and prehospital settings where manual ventilation remains highly operator-related.

### 4.3. Improved Agreement in Intubated Scenarios: Implications for Advanced Airway Management

In contrast to mask ventilation, strong correlations were observed between the test device and simulator measurements during ET-tube (r = 0.854) and I-gel (r = 0.709) ventilation. This pattern is consistent with prior observations that secured airway devices, such as endotracheal tubes and supraglottic airway devices, improve ventilation efficacy and reduce leakage compared with face masks, resulting in more consistent delivery of tidal volumes [17]. Studies using cadaver and simulation models have shown that tidal volumes and airway pressures differ significantly between face mask, supraglottic airway, and endotracheal tube techniques, with intubated airways demonstrating higher expiratory tidal volumes and lower leak (VTi-VTe) values than mask or SGA ventilation, indicating more effective and consistent ventilation when a secure airway is established [18]. Endotracheal intubation provides a closed respiratory circuit with minimal leakage, allowing flow-based sensors to more accurately reflect lung-delivered tidal volume. Supraglottic airway devices, while not as definitive as ETTs, still offer substantially improved sealing compared with face masks [19]. The slightly lower correlation observed with I-gel compared to ETT may reflect residual periglottic leak or positional variability inherent to supraglottic devices. From a clinical perspective, these results suggest that tidal volume monitoring devices may be particularly valuable during advanced airway management, such as during prolonged resuscitation, interfacility transport, or mechanical ventilation bridging. In these contexts, relative agreement and trend monitoring may be more clinically relevant than absolute precision.

### 4.4. Operator-Related Variability and the Role of Experience

A key contribution of this study lies in its examination of operator characteristics as modifiers of measurement discrepancy. Statistically significant main effects were observed for gender, age group, and clinical experience level; however, these findings should be interpreted cautiously given the small and unbalanced subgroup sizes. Interestingly, participants with intermediate experience demonstrated lower discrepancies than both beginners and experts. This non-linear relationship has been reported in other procedural skill studies and may reflect a balance between technical attentiveness and avoidance of overconfidence [20]. Experienced providers may rely more heavily on muscle memory and subjective judgment, potentially leading to greater variability in manual ventilation patterns. The apparent gender-related differences observed in this study should be interpreted with considerable caution. Because the subgroup sample sizes were small and not balanced, these findings should not be interpreted as evidence of intrinsic sex-related differences in ventilation performance. Instead, they likely reflect random variation or differences in individual technique and training background within this limited sample. Importantly, multiple studies have demonstrated that real-time audiovisual feedback during ventilation or CPR significantly reduces inter-operator variability and improves adherence to guideline-recommended ventilation parameters, regardless of provider experience or background [21]. These findings support the interpretation that the discrepancies observed in the present study are modifiable through structured training and feedback, reinforcing the educational and standardizing potential of the tidal volume monitoring device evaluated here. Therefore, the subgroup analyses in the present study should be viewed primarily as exploratory assessments of potential operator-related variability rather than definitive evidence of demographic performance differences. Larger studies will be required to determine whether such factors meaningfully influence ventilation performance when using tidal volume monitoring systems.

### 4.5. Clinical and Educational Implications

Manual ventilation remains a fundamental intervention in prehospital and emergency airway management, yet both observational and simulation studies have consistently shown that delivered tidal volumes and ventilation rates frequently deviate from guideline-recommended targets during out-of-hospital cardiac arrest (OHCA) resuscitation and simulated scenarios. In prospective observational data from real OHCA patients, median expiratory tidal volumes were consistently below recommended guideline targets, and substantial leakage was common during bag-valve-mask ventilations performed by Basic Life Support teams, emphasizing the challenges in achieving guideline-consistent manual ventilation in the field [22]. Given that both hypoventilation and hyperventilation can be associated with poor physiological outcomes, improving adherence to guideline targets for tidal volume and rate is a persistent priority in resuscitation practice. Real-time ventilation feedback systems, such as differential pressure-based or flow sensor-based monitors, are designed to provide immediate visual cues about ventilation volume and rate, facilitating on-the-spot corrections. In a randomized controlled simulation trial of paramedics during simulated OHCA, real-time ventilation feedback significantly increased the proportion of ventilations within guideline-recommended rate and tidal volume targets compared with standard practice without feedback [23]. Similarly, in a simulated cardiac arrest setting using a visual feedback interface, the percentage of ventilations within target for both rate and volume improved markedly when feedback was provided compared with no feedback [24]. These results suggest that devices capable of delivering real-time ventilation data may attenuate operator-related variability, reduce both hyperventilation and hypoventilation, and support adherence to established resuscitation guidelines. In turn, incorporation of such technology into EMS provider training and competency assessments (for example, by providing immediate performance feedback during skills sessions) could enhance learning curves, reinforce correct techniques, and improve retention of proper ventilation skills across clinical experience levels. However, it should be emphasized that the present findings are derived from a controlled simulation environment involving a relatively small number of operators. Although the participants represented healthcare providers experienced in airway management, the sample size was intentionally selected for an initial simulation-based engineering evaluation and may not fully capture the variability of manual ventilation techniques encountered among broader clinical populations. Consequently, although exploratory subgroup analyses were performed to investigate the potential influence of participant characteristics, the study was not statistically powered to detect subgroup differences or interaction effects. In particular, the unequal sex distribution among participants may have influenced these exploratory analyses and limits the generalizability of subgroup-specific findings. Larger studies with more balanced participant populations will therefore be required to confirm these observations. Furthermore, although the three airway management scenarios evaluated in this study represent clinically relevant ventilation conditions, the manikin-based model cannot fully reproduce the wide range of physiological and respiratory mechanical variations encountered in actual patients, including differences in lung compliance, airway resistance, mask leakage, and spontaneous respiratory interactions. These factors may substantially alter airflow dynamics during manual ventilation and consequently influence the agreement and robustness of flow-based tidal volume estimation. Therefore, the performance of the proposed monitoring system observed under standardized laboratory conditions may differ in patients with heterogeneous respiratory mechanics. In addition, the respiratory monitoring system incorporated into the simulation manikin served as a standardized comparative measurement system rather than a certified laboratory reference instrument. Therefore, the present study should be interpreted as an agreement study comparing two independent measurement systems under standardized simulation conditions, rather than as a formal validation of absolute measurement agreement. Accordingly, the present study was intentionally designed as an initial simulation-based evaluation to assess measurement agreement and operational reliability under standardized ventilation conditions before undertaking more comprehensive engineering and clinical validation. Because all experiments were conducted using the same factory-calibrated device under identical laboratory conditions within the manufacturer’s specified operating environmental range, additional calibration before each experimental session was not performed, and environmental factors such as temperature, humidity, and atmospheric pressure were not independently investigated. Under these controlled conditions, the influence of environmental variation on measurement performance was considered minimal. Nevertheless, calibration drift was not independently evaluated during the repeated experimental sessions, and future engineering validation should include longitudinal assessment of calibration stability as well as evaluation under varying environmental conditions. Although simulation studies provide valuable preliminary insights into device performance and usability, further validation against certified laboratory reference systems, such as a calibrated pneumotachograph or spirometer, as well as evaluations under variable respiratory mechanics and validation in real patient settings, will be necessary to establish the absolute measurement agreement, robustness, clinical effectiveness, and translational applicability of the proposed monitoring system.

### 4.6. Future Engineering Development

The findings of the present study provide several directions for future engineering development of real-time tidal volume monitoring systems for manual ventilation. First, further optimization of the sensor calibration procedure and signal-processing algorithm is warranted to reduce the systematic measurement bias identified in this study. Second, future investigations should validate the proposed system against certified laboratory respiratory measurement instruments, such as calibrated pneumotachographs or spirometers, to establish absolute measurement agreement under standardized conditions. Third, device performance should be evaluated under a broader range of simulated respiratory conditions, including varying lung compliance, airway resistance, and leakage scenarios, to improve measurement robustness across diverse ventilation environments. In addition, incorporation of real-time adaptive correction algorithms capable of compensating for dynamic airflow characteristics and mask leakage may further enhance measurement agreement during manual ventilation. Because the clinical value of real-time tidal volume feedback fundamentally depends on the agreement and reliability of the monitored measurements, establishing robust measurement agreement under representative airway conditions should precede large-scale evaluations of clinical effectiveness. Finally, prospective clinical validation involving patients in emergency and prehospital settings will be essential to confirm the clinical utility, reliability, and translational applicability of the proposed monitoring system.

## 5. Conclusions

This simulation-based study demonstrated that a newly developed tidal volume monitoring device provides consistent and repeatable measurements across multiple airway scenarios, despite systematic overestimation relative to simulator-based reference values. Agreement between the test device and the reference system improved substantially under secured airway conditions, highlighting the importance of airway sealing in flow-based tidal volume assessment. Taken together, these findings suggest that the primary value of the device lies not in serving as a replacement for reference-grade measurement systems, but in reducing operator-related variability and supporting safer ventilation practices through real-time feedback, particularly in dynamic and resource-limited prehospital environments.

## Figures and Tables

**Table 1:** Baseline characteristics of study participants (*n* = 20).

Characteristic	Value
Sex, ***n*** (%)	
Male	5 (25.0)
Female	15 (75.0)
Age (years)	
Average (mean ± SD)	25.95 ± 2.89
Median ([IQR])	25 [24–28]
Age group, *n* (%)	
<25 years	9 (45.0)
25–29 years	8 (40.0)
≥30 years	3 (15.0)
Career experience (months)	
Average (mean ± SD)	36.50 ± 38.46
Median ([IQR])	18.5 [7.5–56]
Clinical experience level (months), *n* (%)	
Beginner (≤12 months)	6 (30.0)
Intermediate (13–60 months)	9 (45.0)
Expert (>60 months)	5 (25.0)
Experience using a manual ventilator, *n* (%)	
Yes	17 (85.0)
No	3 (15.0)
Manual ventilator model name (manufacturer), *n* (%)	
Ambu^®^ Mark IV (Ambu A/S)	17 (100.0)
LSR (Laerdal Medical AS)	1 (5.9)
MR-010 (MOW Medical Co., Ltd.)	1 (5.9)

* SD: standard deviation; IQR: interquartile range.

**Table 2:** Paired *t*-test results comparing tidal volume between the simulator and the test device across airway scenarios (N = 200).

Scenario	Comparative Device (mL)	Test Device (mL)	Mean Difference (mL)	95% CI of Difference [IQR] (mL)	t	*p*-Value
BVM	229.59 ± 86.06	514.20 ± 80.35	284.61	[269.69, 299.53]	37.61	<0.001
ETT	319.27 ± 81.84	398.90 ± 56.35	79.63	[73.40, 85.86]	25.19	<0.001
I-gel	329.21 ± 78.57	428.75 ± 62.86	99.55	[91.75, 107.34]	25.20	<0.001

* BVM: bag-valve mask (normal ventilation), ETT: endotracheal intubation, I-gel: supraglottic airway insertion, CI: confidence interval, IQR: interquartile range. * Note: All *p*-values are based on two-tailed paired-samples *t*-tests.

**Table 3:** Intraclass correlation coefficients (ICC) between the simulator and the test device across airway scenarios (N = 200).

Scenario	ICC (Single Measure, Absolute Agreement)	95% CI (Lower–Upper)	F-Value	df	*p*-Value
BVM	0.025	−0.028 to 0.097	1.421	199	0.007
ETT	0.486	−0.092 to 0.789	8.881	199	<0.001
I-gel	0.350	−0.095 to 0.678	5.486	199	<0.001

* BVM: bag-valve mask (normal ventilation), ETT: endotracheal intubation, I-gel: supraglottic airway insertion, CI: confidence interval. * Note: ICC values represent single-measure intraclass correlation coefficients calculated using a two-way mixed-effects model with an absolute agreement definition (ICC(A,1)). Values are presented together with their 95% confidence intervals.

**Table 4:** Summary of Mixed Repeated Measures ANOVA for Measurement Error.

Source	Sum of Squares	df	Mean Square	F	*p* Value	ηp2
Between-Subjects Effects						
Gender	230,146.98	1	230,146.98	46.17	<0.001	0.195
Age Group	50,128.26	2	25,064.13	5.03	0.007	0.050
Clinical experience level	73,868.67	2	36,934.34	7.41	<0.001	0.072
Error	952,128.32	191	4984.97			
Within-Subjects Effects						
Scenario (Time)	2,731,020.28	1.49 *	1,839,405.92	431.40	<0.001	0.743
Scenario × Gender	280,531.95	1.49 *	188,944.82	44.31	<0.001	0.189
Scenario × Age Group	8672.39	2.97 *	2920.53	0.69	0.560	0.007
Scenario × Clinical experience level	58,190.42	2.97 *	19,596.30	4.60	0.004	0.046
Error (Scenario)	1,209,141.55	283.58 *	4263.80			

* Note: Degrees of freedom and *p* values are Greenhouse–Geisser corrected due to violation of the sphericity assumption (χ^2^ = 80.987, *p* < 0.001). ηp2 = partial eta-squared.

**Table 5:** Bonferroni Post-Hoc Comparisons for Age Group and Clinical experience level.

Comparison	Mean Difference	Standard Error	*p* Value
Age Group			
<25 vs. 25–29	24.05	6.26	<0.001
<25 vs. ≥30	18.38	8.73	0.110
25–29 vs. ≥30	−5.68	8.59	1.000
Clinical experience level			
Beginner vs. Intermediate	−28.11	6.79	<0.001
Beginner vs. Expert	−8.12	7.81	0.899
Intermediate vs. Expert	19.99	7.19	0.018

* Note: *p* < 0.05. Bonferroni adjustment for multiple comparisons applied.

## Data Availability

The raw measurement data generated and analyzed during the current study are available from the corresponding author upon reasonable request. Additional information regarding device verification and calibration procedures may also be provided upon reasonable request, subject to institutional and commercial restrictions. The embedded software and signal-processing algorithm of the proposed monitoring system constitute proprietary intellectual property of the manufacturer and are therefore not publicly available.
